# α-Linolenic acid prevents hepatic steatosis and improves glucose tolerance in mice fed a high-fat diet

**DOI:** 10.6061/clinics/2018/e150

**Published:** 2018-10-25

**Authors:** Natália Bonissi Gonçalves, Rafael Ferraz Bannitz, Bruna Ramos Silva, Danielle Duran Becari, Carolina Poloni, Patrícia Moreira Gomes, Milton Cesar Foss, Maria Cristina Foss-Freitas

**Affiliations:** Departamento de Medicina, Divisao de Endocrinologia e Metabolismo, Faculdade de Medicina de Ribeirao Preto, Universidade de Sao Paulo, Ribeirao Preto, SP, BR

**Keywords:** alpha Linolenic Acid, Mice, Glucose Tolerance, Insulin Resistance, Hepatic Steatosis, Inflammation, Endoplasmic Reticulum Stress

## Abstract

**OBJECTIVES:**

Dietary omega-3 fatty acids have been efficacious in decreasing serum cholesterol levels and reducing the risk of cardiovascular disease. However, the metabolic and molecular changes induced by the omega-3 fatty acid α-linolenic acid (ALA), which is found in linseed oil, are not fully understood. In this study, we showed a correlation between ALA and insulin resistance, inflammation and endoplasmic reticulum stress (ERS).

**METHODS:**

We studied 40 male mice (C57/BL6) divided into 4 groups: a control (C) group, a control + omega-3/ALA (CA) group, a high-fat diet (HFD) (H) group and a high-fat diet + omega-3/ALA (HA) group. For 8 weeks, the animals in the H and HA groups were fed a high-fat (60%) diet, while the animals in the C and CA groups received regular chow. The diets of the CA and HA groups were supplemented with 10% lyophilized ALA.

**RESULTS:**

ALA supplementation improved glucose tolerance and reduced insulin resistance, as measured by intraperitoneal glucose tolerance tests and the homeostasis model assessment for insulin resistance, respectively. In addition, ALA reduced hepatic steatosis and modified the standard fat concentration in the liver of animals fed an HFD. Dietary ALA supplementation reduced the serum levels of interleukin 6 (IL-6), interleukin 1 beta (IL-1β) and monocyte chemoattractant protein-1 (MCP-1), increased the expression of important chaperones such as binding immunoglobulin protein (BIP) and heat shock protein 70 (HSP70) and reduced the expression of C/EBP-homologous protein (CHOP) and X-box binding protein 1 (XBP1) in hepatic tissues, suggesting an ERS adaptation in response to ALA supplementation.

**CONCLUSIONS:**

Dietary ALA supplementation is effective in preventing hepatic steatosis; is associated with a reduction in insulin resistance, inflammation and ERS; and represents an alternative for improving liver function and obtaining metabolic benefits.

## INTRODUCTION

Obesity has become an important global public health problem resulting in an increased prevalence of chronic diseases such as type 2 diabetes, metabolic syndrome and coronary heart disease 1. A high-fat diet (HFD) is used as a model to induce obesity and/or insulin resistance by inducing metabolic complications and increased inflammation 2-4. Hepatic steatosis is a metabolic complication that is associated with an HFD 4 and has a high global incidence.

Recent studies have shown that HFD-induced obesity results in a state of chronic inflammation characterized by the infiltration of macrophages, which produce proinflammatory cytokines in adipose tissue 5, 6. Two of the most studied proinflammatory cytokines are tumour necrosis factor-alpha (TNF-α) and interleukin 1 beta (IL-1β), both of which reduce insulin sensitivity through interference with the insulin signalling pathway 7.

In addition to the negative effects on insulin production and signalling processes, HFD-induced inflammation and obesity lead to the overload of various vital organelles 8. Obesity induces ERS, and ERS, in turn, plays a central role in the development of insulin resistance and diabetes 9.

A growing number of individuals across the globe are above a healthy weight; this phenomenon can be explained by an increased consumption of foods rich in fat and low in fibre 10,11. However, the demand for nutrients that show pharmacological actions effective against inflammation and metabolic disorders, including the omega-3 fatty acid family, has grown 12. The most well-known polyunsaturated fatty acids are the n-3 (omega-3) and n-6 (omega-6) fatty acids, which have distinct and opposite metabolic functions. The polyunsaturated n-6 fatty acids (n-6 PUFA) promote inflammation, while the n-3 polyunsaturated fatty acids (n-3 PUFA) inhibit the inflammatory process 13. Several studies have shown that the omega-3 fatty acids eicosapentaenoic acid (EPA) and docosahexaenoic acid (DHA) can decrease inflammation, but little is known about the effect of the polyunsaturated fatty acid omega-3 α-linolenic acid (ALA).

On the other hand, fatty acids are known to exert some of their beneficial systemic effects on hepatic steatosis by altering the fatty acid profile of phospholipids present in hepatic tissue 14. In addition, a diet rich in ALA can prevent alcoholic liver disease by improving liver lipid homeostasis in animal models 15.

ALA is present in large proportions in linseed oil and is a precursor of eicosapentaenoic acid (20:5n-3) and docosahexaenoic acid (20:6n-3), which are associated with the prevention of cardiovascular diseases 16.

Thus, to more clearly understand the role of ALA, which is found in linseed oil, in the mouse diet, we used an animal model of insulin resistance induced by a diet high in fat and demonstrated that in this animal model, the supplementation of ALA leads to a reduction in both insulin resistance and inflammation and affects ERS by increasing the production of important chaperones.

## MATERIALS AND METHODS

### Animal Procedures and Diets

We studied 40 6-week-old male mice (C57/BL6) obtained from the animal facilities of the School of Medicine of Ribeirao Preto, São Paulo University, Brazil. The investigation was approved by the local ethics committee on animal research and followed the Guide for the Care and Use of Laboratory Animals published by the U.S. National Institutes of Health (NIH publication no. 85-23, revised 1996). The animals were maintained on a 12h:12h artificial light-dark cycle and were housed 5 animals to a cage. The animals were randomly divided into four groups: control (C), control/ALA (CA), high-fat diet (H) and high-fat diet/ALA (HA). The mean weight of the mice was 14.1 g. The animals in the control group received standard chow (3,710 kcal/kg; Nuvilab CR1, São Paulo, Brazil), while those in the CA group received standard chow supplemented with 10% lyophilized ALA from flaxseed, for a period of 8 weeks. The animals in the H group received a manipulated HFD (approximately 60% fat), while those in the HA group received the high-fat diet supplemented with 10% lyophilized ALA from flaxseed, for a period of 8 weeks. In all groups, the diets and water were provided ad libitum. Detailed nutritional information for all the diets used in the study is shown in Tables 1 and 2. After the period of supplementation, the animals were euthanized by decapitation and were dissected. Liver tissues were weighed, flash-frozen in liquid nitrogen, and stored at -70°C until further processing. Some liver tissue samples were fixed and processed for histopathological analysis; in addition, blood was collected for the quantification of serum glucose, lipid, insulin and cytokine. The animals were evaluated every four weeks, and body weight and glucose measurements were taken after fasting for 6 hours.

### Intraperitoneal Glucose Tolerance Test

One week before being euthanized, all the animal groups were subjected to intraperitoneal glucose tolerance tests (IPGTTs). The test was conducted after 6h of fasting, and 2 g of glucose/kg body weight was administered intraperitoneally to each animal. Blood glucose was determined from the tail vein using a glucometer (Accu-Chek, Roche, Brazil), which monitored the level of glucose at 0, 30, 60, 90 and 120 minutes. Time versus glucose level was plotted for each animal to calculate the area under the curve (AUC).

### Quantification of Serum Glucose, Lipids, Insulin and Cytokines

To determine the serum glucose levels of animals fed different diets, we measured the blood sugar levels of all the mice after 6 weeks of treatment. Blood was centrifuged for 10 min at room temperature; plasma was collected and stored at -70°C. Serum glucose was measured using a glucometer. Serum and liver lipid fragments, cholesterol, and triglycerides were analysed using commercial kits (Labtest Diagnóstica S.A., Brazil).

To evaluate the inflammatory process in relation to dietary ALA supplementation, we measured the levels of the main interleukins responsible for inflammation. Serum cytokine levels were measured using an Enzyme Linked Immunosorbent Assay (ELISA) Kit Mouse Cytokine/Chemokine Magnetic Bead Panel (MILLIPLEX^®^ Map, Luminex^®^ Technology) (Merck, Damstadt, Germany). Insulin levels were measured using an Ultrasensitive ELISA Kit (DRG Diagnostics, Marburg, Germany).

### HOMA Index Determination

The homeostatic model assessment index was calculated from the plasma glucose and insulin levels in blood samples after 6 hours of fasting. Blood glucose was measured in whole blood using a glucose meter (Accu-Chek), and insulin was measured using an Ultrasensitive ELISA Kit (DRG Diagnostics, Marburg, Germany). The HOMA IR index was calculated using the following formula: [glucose (mmol/L) × insulin (µU/L) / 22.5]. The HOMA β index was calculated using the following formula: [20 × insulin (µU/L) / glucose (mmol/L) - 3.5].

### Total Liver Fat

To evaluate the concentration of fat in the liver of animals supplemented with ALA, we quantified the total fat present in hepatocytes and conducted a histological study to detect the presence of fat droplets in the hepatic tissues. Total liver fat was determined according to the method published by Bligh and Dyer in 1959 17. Briefly, this method is characterized as a cold method that uses a mixture of chloroform, methanol and water. The sample was triturated with methanol and chloroform, leaving only one phase. Then, additional chloroform and water were added, establishing two phases. The upper phase was removed, and after evaporation, the result was calculated as the difference in the final weight of the beaker compared with the initial weight.

### Histopathological Analyses

Liver biopsy tissues were fixed in a 5% formaldehyde-buffered solution for 24 hours and embedded in paraffin for subsequent histological processing. Sections of 5 μm in thickness were stained with haematoxylin and eosin. The stained slides were analysed using a Leica^®^ DM4000B microscope with a Leica^®^ DFC 280 camera connected to a computer with LAS^®^ - Leica Application Suite (version 3.3.0) software for image capture.

### Protein Extraction and Western Blot Analysis

For protein extraction, 1 mL of ice-cold RIPA buffer (30 mM HEPES, 150 mM NaCl, 10% glycerol, 1% Triton X-100, 0.5% sodium deoxycholate and 1 ml of protease inhibitor per litre) was added to 100 g of frozen liver tissue, and the tissue was homogenized with a Polytron mixer in an ice bath. The liver tissue homogenates were incubated for 2h at 4°C with gentle shaking. Then, the homogenates were centrifuged at 12,000 rpm for 20 minutes at 4°C, and the supernatants were transferred to clean tubes. Total protein concentrations were determined using a BCA kit. Protein samples were diluted in sample buffer, incubated at 95°C for 10 min and separated by electrophoresis using 10% acrylamide SDS gels. The proteins were transferred to nitrocellulose membranes by semidry transfer (Trans-Blot, Bio-Rad) at 25 V for 30 minutes. For Western blotting, the membranes were blocked with TBS-T (1× Tris-buffered saline + 0.05% Tween 20) in 5% skim milk and were then incubated overnight with primary antibodies diluted 1:1000. After being washed, the membranes were incubated for 1 hour at room temperature with anti-rabbit antibodies diluted 1:3000. After the final wash with TBS-T, the blots were developed using an HRP substrate in the Chemi-Doc XRS+ System (Bio-Rad). Bands were analysed using Image Lab software (Bio-Rad).

### Materials

Nitrocellulose paper (#1620177), Laemmle sample buffer (#1610737) and HRP substrate (IMMUN-STAR #1705040) were from Bio-Rad (Hercules, CA, USA). The Bicinchonic Acid Protein Assay Kit (#BCA1; B9643) and protein inhibitor cocktail (#P2714) were from Sigma Chemical Co. (St. Louis, MO). The anti-heat shock protein 70 (HSP-70) (rabbit polyclonal, SC-71060R), anti-C/EBP-homologous protein (CHOP) (rabbit polyclonal, SC-575) and anti-X-box binding protein 1 (XBP-1) (M186) (rabbit polyclonal, SC-7176) antibodies were from Santa Cruz Biotechnology, Inc. (Dallas, Texas, USA). The anti-glucose regulating protein 78 (BIP) (rabbit polyclonal, #C50B12) antibody was from Cell Signaling Technology (Beverly, MA, USA). Routine reagents were purchased from Sigma Chemical Co. (St. Louis, MO, USA).

### Statistical Analyses

The data were tabulated and analysed using Graph Pad Prism^®^ for Mac OS X, version 5.0c. The results are presented as the means ± standard deviation with median intervals between quartiles where appropriate. We used ANOVA to compare the results among the four groups, and we used *Student's t-test* to compare the results between two groups. The level of significance adopted was *p*<0.05 (5%).

## RESULTS

### Food consumption and weight

The consumption of chow was investigated so that an ad libitum environment could be provided. Even though the source of omega-3 fatty acids was not fish oil but linseed oil, the diet was well tolerated, and the food supply was exhausted in approximately 18 hours. Therefore, a daily intake of 3 g of ALA was ensured for all the animals in the groups.

**Figure 1:**
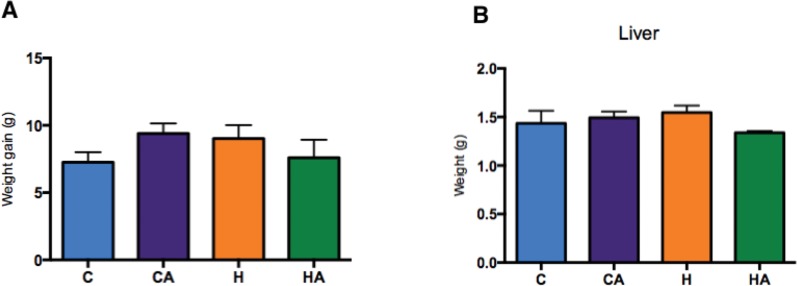
**Body and liver weights measured during the study** Weight gain (A) and liver weight (B) were similar between the groups by the end of the supplementation period in animals fed a chow diet or an HFD diet supplemented with ALA (3 g of ALA/day) (CA and HA) or diets not supplemented with ALA (C and H). One-way ANOVA (Tukey's *post hoc* test): *p*=0.39 and *p*=0.27, respectively. Data are presented as the means ± s.e.m.

We observed that the animals of groups C and CA had a body weight gain of 7.2 (±1.5) g and 9.4 (±1.7) g, respectively, while groups H and HA presented a body weight gain of 9.0 (±2.0) g and 8.6 (±3.0) g, respectively (Figure 1A); however, the weight of the liver, corrected for the total body weight, did not increase in the animals from the HA group as they did in the H group. Instead, the corrected liver weight of the HA group showed a median close to that of the C and CA groups, although no significant differences were observed (Figure 1B). We also noticed that there was no significant difference in food intake, food efficiency and energy efficiency among the groups (Table 3).

### ALA supplementation decreased the glucose level

**Figure 2:**
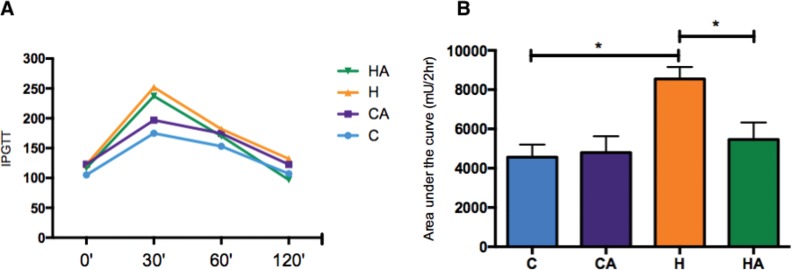
**Glucose levels and area under the curve for the IPGTT.** A) Glucose levels during the IPGTT. HA, high-fat diet + 10% ALA; H, high-fat diet; CA, control chow +10% ALA; C, control chow. B) Area under the curve for the IPGTT. Student’s t-test: *p<0.05. *p*=0.0264.

Animals fed the HFD had a decreased glucose tolerance, as depicted in the IPGTT graph (Figure 2A). ALA supplementation induced a significant improvement in glucose tolerance in the HA group compared with that in the H group, and there was no difference in glucose tolerance between the groups of animals fed the control diet (Figure 2B).

**Figure 3:**
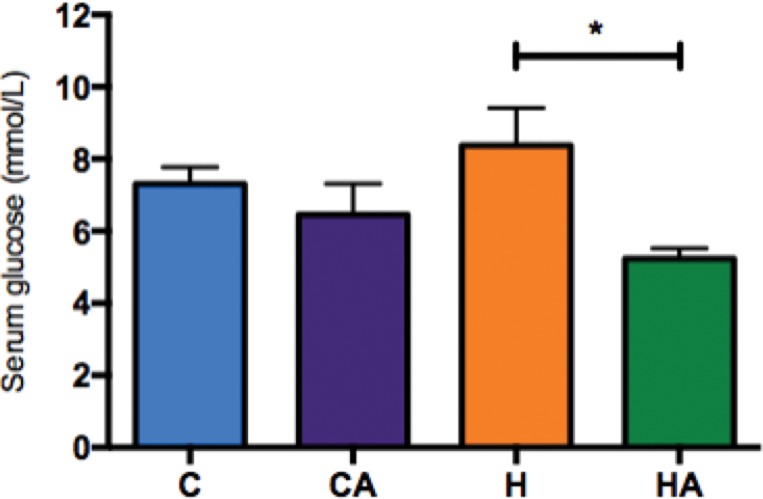
**Glucose levels after 8 weeks of treatment.** Serum glucose after 8 weeks. HA, high-fat diet + 10% ALA; H, high-fat diet; CA, control chow +10% ALA; C, control chow. One-way ANOVA (Tukey’s *post hoc* test): *p=0.0448.

Animals supplemented with ALA had decreased blood glucose levels. This outcome was especially true in the comparison of the HA group with the H group (Figure 3A). We measured serum insulin levels but found no differences between the groups (Supplementary Figure 1). We observed a lower HOMA IR index in the HA group than in the H group (0.1±0.06 and 0.2±0.16, respectively, p=0.04), and no differences were observed in the HOMA β index (ANOVA, *p*=0.69, Table 3).

### ALA affects the distribution of fat in the liver

**Figure 4:**
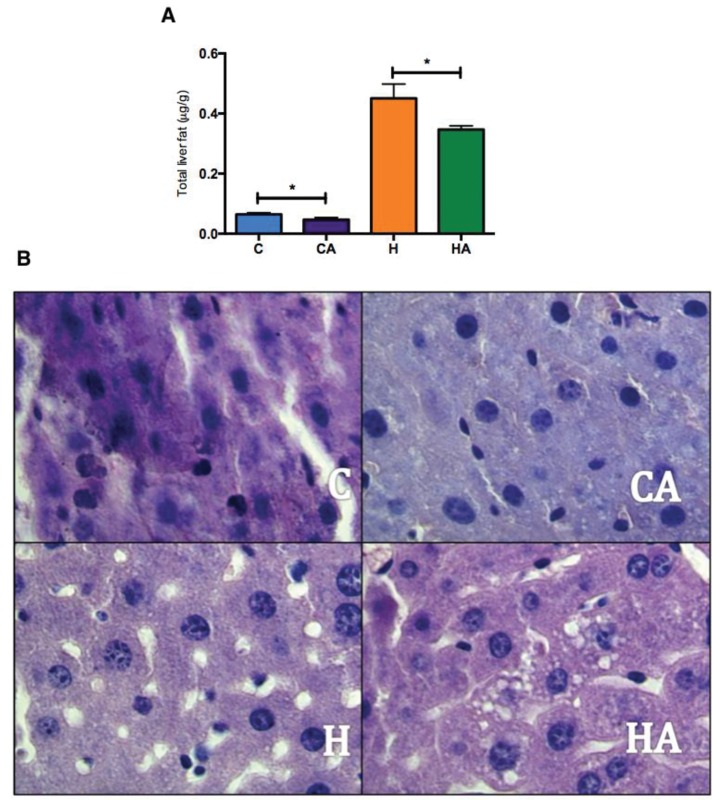
**Total liver fat levels and histopathological images of liver tissue** A) Total liver fat (μg/g). One-way ANOVA (Tukey’s *post hoc* test): *p<0.0001; B) Histopathological images of liver tissue. HA, high-fat diet + 10% ALA; H, high-fat diet; CA, control chow +10% ALA; C, control chow. Magnification 1000×.

We noted that the animals treated with ALA (CA and HA groups) exhibited less total fat in the liver than did the animals without ALA supplementation (Figure 4A). Additionally, by histopathological tissue analysis (Figure 4B), we found that the liver fat concentrations were higher in the H and HA groups than in the C and CA groups. The total fat concentration was lower in the liver of the animals supplemented with ALA (CA and HA groups) than in the liver of the other animals, and histopathological analysis showed both a decrease in the size of the lipid droplets and altered fat distribution, with focal areas of fat concentrated in the liver tissue. Therefore, these data show that omega-3/ALA supplementation is effective in preventing hepatic steatosis.

Even though our data indicated a reduction in hepatic fat, the serum cholesterol levels were increased in the animals supplemented with ALA (Supplementary Figure 2A); however, the triglyceride levels were not different between the groups (Supplementary Figure 2B).

### ALA ameliorates the inflammatory profile

**Figure 5:**
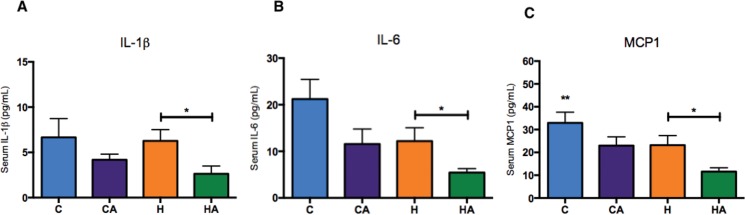
**Inflammatory profiles** A) IL-1β (pg/ml) serum levels. Student’s t-test: *p=0.0417; B) IL-6 (pg/ml) serum levels. Student’s t-test: *p=0.0445; C) MCP-1 (pg/ml) serum levels. Student’s t-test: *p=0.0348. HA, high-fat diet + 10% ALA; H, high-fat diet; CA, control chow +10% ALA; C, control chow.

Serum IL-1β, interleukin 6 (IL-6) and monocyte chemoattractant protein-1 (MCP-1) were significantly reduced in the HA group compared with those in the H group (Figure 5A-C), indicating that ALA supplementation in animals fed an HFD prevented the inflammatory process, which is a characteristic outcome of a high-fat diet. We also evaluated TNF-α levels, but we found no differences between the groups (Supplementary Figure 3).

### ERS decreases with ALA administration

**Figure 6:**
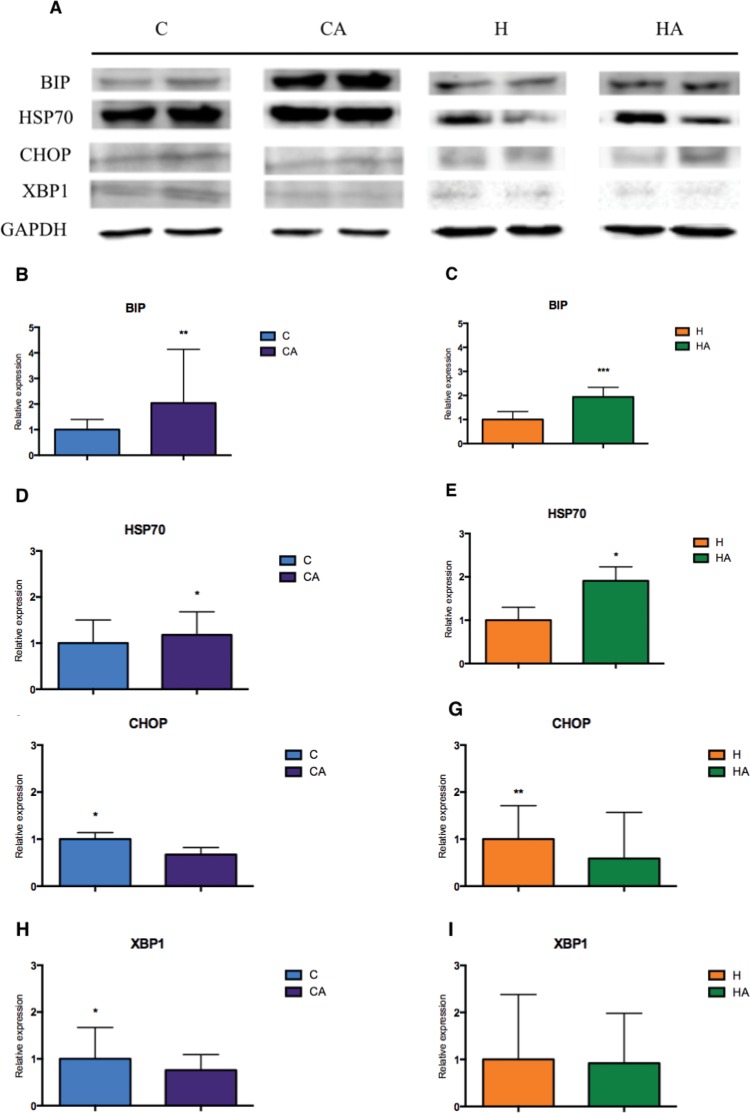
**Evaluation of proteins involved in the activation of the endoplasmic reticulum stress response in the C, CA, H and HA groups.** A) Immunoblotting of different proteins evaluated in liver tissues from animals fed regular chow, an HFD, or an HFD supplemented with 10% omega-3/ALA. GAPDH was used as a control and for normalization of the results. Relative quantification of the expression levels of BIP (B) *p*=0,0054, HSP70 (C) *p*=0.0194, CHOP (D) *p*=0.0354, and XBP1 (E) *p*=0.0194, between the C and CA groups; relative quantification of the expression levels of BIP (F) *p*=0.0002, HSP70 (G) *p*=0,0311, CHOP (H) *p*=0.0077, and XBP1 (I) *p*=0.6971, between the H and HA groups; Student’s t-test: *p<0.05, **p<0.01; ***p<0.005.

With the intention of studying the effects of ALA in relation to ERS, we evaluated the levels of the main proteins involved in the unfolded protein response (UPR) in the liver. The expression of both binding immunoglobulin protein (BIP [HSPA-5]) and the 70 kilodalton heat shock protein (chaperone HSP70) increased in animals supplemented with ALA (CA and HA groups, Figure 6B-E). However, the expression of CHOP and X-box binding protein 1 (XBP1), key proteins in ERS, were decreased by ALA supplementation in the CA and HA groups (Figure 6F-I). These data corroborate each other, demonstrating that ALA supplementation in animals fed an HFD inhibits the inflammatory process and can decrease ERS, thus ameliorating the characteristic effects of an HFD.

## DISCUSSION

Many studies are trying to understand how omega-3 fatty acids, especially EPA and DHA of animal origin, can fight diseases related to metabolic syndrome. Our group sought to understand how the supplementation of omega-3 fatty acids from linseed (*Linum usitatissimum*), the richest source of ALA, interfered with metabolism, inflammation, and ERS. ALA may be converted in the body to EPA and DHA 18,19. Since this type of omega-3 fatty acid is more accessible to the global population, we used a known concentration of flaxseed extract so that our findings could easily be applied to people's daily diets by means such as increasing the daily consumption of plant sources rich in ALA.

Although no difference in body weight gain was observed between the groups fed control chow and an HFD, as expected, this treatment induced the onset of typical phenotypes of metabolic syndrome. The absence of a greater body weight gain in mice fed an HFD has been previously observed 20. The use of diets that contain high amounts of lipids for prolonged time periods causes a decrease in the amount of energy consumed by the animals during the weeks of treatment 21. We showed not only that an HFD significantly increased the amount of fat infiltration in the liver but also that ALA supplementation altered the pattern of steatosis by modifying the distribution of vesicular macrophage infiltration. The reduction in hepatic steatosis observed in our study was reinforced by the histopathological analysis, which clearly showed the change in the pattern of distribution of lipids in the hepatocytes. This pattern change was confirmed in other studies, which showed the same redistribution in animals that were subjected to an HFD supplemented with ALA 22. In fact, there are few animal studies showing that ALA supplementation significantly reduced the amount of lipids in the liver, as observed in our study 22. Interestingly, our study reported similar results to those of fish-oil-based experiments, which suggest that high concentrations of ALA may result in the efficient conversion of EPA and DHA and may reduce the amount of hepatic fat 23. These observations may explain the similarity of these other results compared with our data, thereby demonstrating that ALA is a plant source of omega-3 fatty acids that can effect efficient metabolic changes.

The animals fed an HFD supplemented with ALA had a decreased concentration of lipids and a redistribution of fat in their liver cells, which reduced hepatic steatosis.

As proposed by Marchesini et al., fat accumulation in liver tissues is a result of high levels of free triglycerides in the serum that consequently induce insulin resistance and increase blood glucose levels; thus, the reduction in steatosis shown by our data is responsible for improving the metabolic profile 23.

To trace evidence of insulin resistance, we analysed the AUC of the IPGTT and HOMA IR curves. Compared with animals fed the control diet, animals fed an HFD showed a significant increase in glucose intolerance, resulting in a higher AUC. However, when the H group was compared with the HA group, we observed an improvement in glucose tolerance associated with a reduction in insulin resistance, which was confirmed by the HOMA IR values.

Various researchers have shown that hepatic steatosis associated with insulin resistance leads to an increase in inflammatory status 24-26. Thus, we explored the effects of dietary ALA and of an HFD on the main inflammatory markers. There is a link between obesity/metabolic syndrome and the immune response; this condition activates inflammatory pathways such as the c-jun N-terminal kinase (JNK) and nuclear factor κB (NFκB) pathways 26. In organisms with both metabolic syndrome and insulin resistance, some inflammatory cytokines, such as TNF-α, IL-6, and MCP-1, are modulated by fatty tissue 26.

Interleukins IL-1β and IL-6 participate in the recruitment of cells, mainly neutrophils, macrophages, NK cells and lymphocytes favouring the Th1 profile, to the inflammatory site. MCP-1 is responsible for the recruitment of monocytes and is related mainly to the atherosclerotic process by recruiting M1 macrophages, which favour the maintenance of inflammation 27.

Given the evidence that high levels of inflammatory markers secondary to obesity may be responsible for the development of comorbidities, many researchers believe that improvement in the inflammatory profile, such as that promoted by weight loss, can consistently reduce the risk of diseases associated with metabolic syndrome, mainly by reducing the levels of these markers 28.

Considering the already well-known anti-inflammatory effect of omega-3 fatty acids, we observed a reduction in key inflammatory markers in animals that were fed an HFD supplemented with ALA. This finding reinforces the hypothesis that the effect of ALA is especially efficient under conditions of caloric overload. The contribution of ALA to the reduction in MCP-1, IL-1β, and IL-6 levels in this study has been confirmed by other studies using ALA supplementation in rats and cell culture 29, 30. Together, these results are of great importance when considering the role of ALA in controlling the inflammatory response.

In addition to the inflammatory processes promoted by obesity, the increase in visceral adipose tissue particularly leads to stress in several vital organelles, especially the endoplasmic reticulum (ER) 8. Obesity and insulin resistance induce a process called endoplasmic reticulum stress (ERS) that results in significant dysfunction of the ER. ERS can be caused by the accumulation of unprocessed proteins within the ER, and in response, ERS starts a broader process called the unfolded protein response (UPR). Activation of the UPR initiates a series of adjustment mechanisms seeking to restore cellular homeostasis, including a decrease in the transfer of proteins into the ER and an increase in the synthesis of chaperone proteins that both neutralize folded proteins until they are processed and increase the transport of folded proteins into the cytoplasm, where they are degraded by proteasomes 31. Acknowledging the importance of ERS, we sought to understand whether the supplementation of ALA could modulate ERS. Our results demonstrated that animals supplemented with ALA, both those fed a control diet and those fed an HFD diet, showed significantly increased levels of BIP and HSP70, which are chaperone proteins that have important functions in controlling ERS. Increased levels of BIP or heat shock protein 5 (HSP5) and HSP70 are associated with lower insulin resistance and reduced hepatic steatosis 26, 32. We also found that the use of ALA modulated the expression of CHOP and XBP1, which are proteins that directly affect ERS. The reduction in CHOP expression, observed both in animals fed the control diet and in those fed the HFD diet supplemented with ALA, demonstrated the effect of ALA on CHOP, leading to a reduction in caspase-mediated apoptosis in the organisms 33.

Our data are consistent with other studies reporting that ALA may play a protective role in ERS by significantly reducing the expression of CHOP 27, 29. In addition, ALA modulated the expression of XBP1, a protein that plays a key role in activating the ER stress response and in controlling glucose and lipid metabolism 9, 30.

In summary, our data showed that ALA supplementation improved glucose tolerance and reduced hepatic steatosis in an animal model of insulin resistance induced by an HFD by modulating mechanisms involved in the development of insulin resistance, such as inflammation and ERS. However, further studies and clinical trials are required to confirm our data and to support plant-derived ALA supplementation for the treatment of steatosis and insulin resistance.

## AUTHOR CONTRIBUTIONS

Gonçalves NB performed the study and analyzed the results. Bannitz RF analyzed the results and drafted the manuscript. Silva BR, Becari DD and Poloni C helped in the care and treatment of animals. Gomes PM performed the IPGTTs. Foss MC provided the space and laboratories for measurements. Foss-Freitas MC coordinated the project and revised the manuscript. All of the authors contributed to the final version of the manuscript.

## Figures and Tables

**Table 1:** Distribution of the macronutrients in and the energy density of the control diets and the high-fat diets.

	Control	Control + ALA	High-fat Diet	High-fat Diet + ALA
Carbohydrates (g/kg)	625.0	503.7	345.0	298.0
Lipids (g/kg)	119.0	288.9	512.5	579.0
Proteins (g/kg)	256.0	207.4	142.5	123.0
Energy density (Kcal/kg)	3710	4610	5619	6519
kcal/day/animal (average)	14.13	24.24	27.5	31.94

Nutritional amounts correspond to the macronutrients (carbohydrates, lipids and proteins) and the energy density contained in the diets: Control, Control +ALA, High-fat Diet and High-fat Diet + ALA.

**Table 2:** Nutritional composition of the control diet and the high-fat diet (weight/100 g).

	Control	High-fat Diet
Carbohydrates	580.61 g	485.82 g
Proteins	237.44 g	200.25 g
Lipids	48.64 g	322.29 g
Fibre	*	*
Iron	50 mg	21.94 mg
Calcium	10.0 g	7.65 g
Phosphorus	8 g	3.1 g
Potassium	*	5321.64 mg
Sodium	2700 mg	2566 mg
Magnesium	*	338.08 mg
Zinc	60 mg	37.58 mg
Manganese	60 mg	24.17 mg
Copper	10 mg	4.36 mg
Folic Acid	6 mg	2.4 mg
Thiamine	14.4 mg	5.9 mg
Riboflavin	11 mg	9.2 mg
Vitamin B12	60 mcg	24 mcg
Vitamin A	25500 Ui	13888 Ui
Vitamin E	60 Ui	34.7 Ui
Cholesterol	*	553.6 mg
Saturated Fatty Acids	*	138.84 g
Monounsaturated Fatty Acids	*	115.73 g
Polyunsaturated Fatty Acids	*	33.95 g

*not established values.

**Table 3:** Metabolic and inflammatory parameters (mean ± SD) of the C, CA, H and HA groups.

	C		CA		H		HA	
	Mean	SD	Mean	SD	Mean	SD	Mean	**SD**
Weight gain (g)	7.2	1.5	9.4	1.7	9.0	2.0	8.6	3.0
Food consumption (g)	2022		2791		2331		2351.5	
Food efficiency (g/g)	0.0035		0.0032		0.0039		0.0037	
Energy consumption (kcal)	7501.6		12866.5		13097.9		15329.4	
Energy efficiency (g/kcal)	0.00095		0.00070		0.00069		0.00056^b^	
Final serum glucose (mmol/L)*	7.30	0.9	6.45	1.9	8.37	1.97	5.23	0.64
Serum insulin levels (pmol/L)*	8.2	2.8	10.7	4.1	4.2	3.8	2.9	2.2
HOMA IR*	0.4	0.14	0.7	0.30	0.2	0.16	0.1	0.06
HOMA β	5.9	1.6	4.2	1.7	3.5	3.4	6.3	6.4
AUC (mU/2 hr)*	4562	1436	4797	1854	8549	1213	5465	1724
Liver weight (g/g of weight)	4.20	0.51	4.21	0.31	4.61	0.21	4.35	0.24
Serum total cholesterol levels (mmol/L)*	2.71	0.21	3.46	0.36	4.80	0.52	6.27	1.17
Serum triglycerides (mmol/L)	1.01	0.29	0.97	0.68	1.50	0.60	1.73	0.53
Serum TNF-α (pg/mL)	3.61	1.2	4.14	1.6	3.47	1.3	2.64	1.5
Serum IL-6 (pg/mL)*	19.58	13.0	11.55	7.2	11.21	7.6	5.44	2.2
Serum IL-1β (pg/mL)*	6.65	5.9	4.18	1.4	6.26	3.3	2.63	2.1
Serum MCP-1 (pg/mL)*	32.92	14.8	22.96	8.7	23.17	11.1	11.55	4.2

Results are expressed as the means and SD. C: control group, fed regular chow; CA: control plus omega-3/ALA group, fed regular chow supplemented with 10% omega-3/ALA; H: high-fat diet group, fed a high-fat diet containing 60% lipids; HA: high-fat diet plus omega-3/ALA group, fed a high-fat diet containing 60% lipids supplemented with 10% omega-3/ALA. TNF-α: *tumour necrosis factor-alpha*, IL-6: *interleukin 6*, IL-1β: *interleukin 1 beta*, MCP-1: *monocyte chemoattractant protein-1*.

*One-way ANOVA (Tukey’s *post hoc* test): *p*<0.05.

*Student’s t-test: *p*<0.05.
